# Tobacco harm perceptions, regulatory attitudes, and cessation intentions before and after the COVID-19 lockdown in California

**DOI:** 10.1177/20551029241306095

**Published:** 2024-12-02

**Authors:** Natalie R Beylin, Anna V Song, Anna E Epperson

**Affiliations:** 1 Department of Psychological Sciences, 33244 University of California, Merced, CA, USA; 2 Nicotine and Cannabis Policy Center, Health Sciences Research Institute, 33244 University of California, Merced, CA, USA

**Keywords:** beliefs, perception, policy, smoking, social cognitions, tobacco control, tobacco prevention, cessation

## Abstract

The present study examined tobacco health perceptions, regulation attitudes, and cessation intentions among California adults before and after the COVID-19 lockdown, given the pandemic’s mixed impact on tobacco use. An online survey of California adults was conducted in two phases: pre-lockdown (March 2020, *n* = 1349) and post-lockdown (May 2020, *n* = 1201). Participants (*M* age 30.29 years; *SD* = 5.91) from both samples were predominately former or current smokers, male, and non-Hispanic White (>60% for all). This method allowed for a comparison of attitudes and behaviors across two distinct periods with two samples. There were significant differences between pre- and post-lockdown risk perceptions, regulatory attitudes, and cessation intentions. Examining shifts in perceptions and attitudes amidst the pandemic aids in understanding the complex and dynamic nature of tobacco behavior change through the lens of a major socioenvironmental event to guide future tobacco control efforts.

## Introduction

The coronavirus (COVID-19) pandemic emerged in early 2020, creating a major health crisis. An infectious respiratory illness caused by a global outbreak of the SARS-CoV-2 virus, COVID-19 has claimed the lives of over 1 million people in the United States thus far (Center for Disease Control, 2022) and was declared the third leading cause of death in 2020 and 2021 (National Institutes of Health, 2022). Meanwhile, the leading cause of preventable death in the United States remains tobacco use (Centers for Disease Control and Prevention, 2021). Per year, over 480,000 people die from cigarette smoking, the most prevalent form of tobacco use, in the United States, and more than 41,000 of those deaths are from secondhand smoke exposure (Centers for Disease Control and Prevention, 2021). In addition, over 16 million adults currently suffer from a smoking-related disease, including cancer, stroke, heart disease, lung disease, diabetes, and chronic obstructive pulmonary disease (Centers for Disease Control and Prevention, 2021).

### Socioenvironmental factors impacting tobacco use

Although over 30 million adults in the United States currently smoke cigarettes (Center for Disease Control, 2022), there has been substantial reduction in tobacco consumption over the past few decades, due in large part to socioenvironmental and policy changes. The movement to regulate false and dangerous cigarette marketing as well as educate the public on the risks of smoking cigarettes created a shift in perception beginning in the late 1960s, gaining continuous momentum ever since (Cummings and Proctor, 2014). Smoking cigarettes developed a negative social stigma and subsequent favorability towards tobacco control efforts and regulatory attitudes increased (Frieden et al., 2005; Gilpin et al., 2004). A series of major policy changes proceeded: smoke free air laws, the banning of smoking inside public establishments, and increasing minimum age for sale of tobacco (Gilpin et al., 2001; Hyland et al., 2012). It is clear that perceptions regarding smoking influences policy and vice versa (Cummings and Proctor, 2014; Stuber et al., 2008). Thus, it is useful to examine changes in general beliefs towards smoking in the context of the COVID-19 pandemic lockdowns–a widespread public health event that held great psychological, social, and economic impact on people’s lives (e.g., illness, socioeconomic effects of the lockdown)–to further guide tobacco control efforts in a changing landscape.

### Rural tobacco-related disparities

Despite the 70% decrease in cigarette smoking over the last 50 years, declines in smoking have not been equal across all groups. In particular, adults living in predominately rural communities are at a higher risk for tobacco use compared to their urban counterparts (Buettner-Schmidt et al., 2019). This could be a result of tobacco control efforts and policies not being tailored to rural populations, limited health services and communication in rural areas, and various demographic and socioenvironmental factors, such as lower income (Buettner-Schmidt et al., 2019; Doogan et al., 2017; Matthews et al., 2017).

The state of California is a primary example of disparities in tobacco use. Overall, around 9% of adults smoke in California, yet smoking rates in the mainly rural Central California region (e.g., San Joaquin Valley, Sierra Foothills) are around 14% (San Joaquin County Public Health Services, 2024). Central California is a racially/ethnically diverse region which faces environmental, socioeconomic, and health-related challenges–such as limited healthcare resources and higher rates of heart disease, asthma, and diabetes (Alcala et al., 2018; Spada et al., 2019). As these medical conditions are risk factors for severe COVID-19 symptoms and outcomes (Alizadehsani et al., 2021), people in Central California might have been (and are) more susceptible to severe COVID-19 symptoms and outcomes, while also lacking the essential resources to stay healthy (Coffman et al., 2017). Given that this large region has higher cigarette smoking rates, health-related challenges, and may be more at risk for severe COVID-19 outcomes, the present study’s population of focus is residents of Central California in an effort to better understand tobacco-related health disparities.

### COVID-19 pandemic and tobacco risk perceptions, attitudes, and behaviors

COVID-19 presents a particular threat to smokers in various ways. First, smoking is a risk factor for many of the medical conditions (heart disease, lung disease, diabetes, cancer, etc.) identified as risk factors for severe COVID-19 illness and COVID-19 related death (Alizadehsani et al., 2021). Second, COVID-19 is a respiratory illness and the Centers for Disease Control and Prevention (CDC) recognizes smoking itself as a medical condition that might worsen COVID-19 symptoms (Centers for Disease Control and Prevention, 2022). Clinical studies examining COVID-19 cases and smoking status suggest that smoking is associated with severe COVID-19 (Gülsen et al., 2020; Karanasos et al., 2020; Patanavanich and Glantz, 2020; Reddy et al., 2021). In previous studies, smokers have reported higher levels of worry towards COVID-19 and perceived susceptibility and severity of COVID-19 (Jackson et al., 2021; Nyman et al., 2021; Yingst et al., 2021) and also indicated higher levels of desire to quit smoking (Klemperer et al., 2020; Yingst et al., 2021).

The relationship between severe COVID-19 illness worry and intentions to quit smoking follows the Health Belief Model (HBM), which suggests perceptions of susceptibility and severity to health risk influences behavior (Rosenstock, 1974). However, although there have been reported higher levels of desire to quit smoking associated with COVID-19 risk perceptions in response to the pandemic (Klemperer et al., 2020; Li et al., 2021; Nyman et al., 2021; Yingst et al., 2021), these cues to action have not actually translated into widescale significant smoking reduction behavior. For example, research by Vogel et al. (2021) and Nyman et al. (2021) indicates that higher COVID-19 risk perceptions were linked to both increases and decreases in smoking behavior among participants. In contrast, Gonzalez et al. (2021) observed a rise in smoking following the lockdown. The divergent findings between Vogel et al. (2021), Nyman et al. (2021), and Gonzalez et al. (2021) could be attributed to variations in their sample populations and methodologies for assessing smoking rates. Vogel et al. (2021) and Nyman et al. (2021) utilized nationally representative samples and gathered data post-lockdown by inquiring participants about perceived changes in their smoking habits since the onset of the pandemic. On the other hand, Gonzalez et al. (2021) based their findings on comparisons of pre- and post-lockdown data regarding smoking occurrences within the past 30 days, focusing on a population of adults from California.

These findings suggest that smoking behavior is far more nuanced than simply perceived risks which is why despite knowledge of the health risks, millions still smoke. More recent health behavior models, such as dual-process theories of decision making, might serve as an explanation as to why stated intentions to quit are not translating into smoking reduction behavior in the context of the COVID-19 pandemic (Gerrard et al., 2008). These theories posit that humans have two thinking processes: a fast, automatic, emotion-based process (*system 1*) and a slow, controlled, reason-based process (*system 2*). In the context of tobacco consumption during the COVID-19 lockdowns, it is possible that smokers’ effortful and conscious plans to quit smoking (*system 2*) were being overshadowed by their rapid judgements (*system 1*). For example, perhaps smokers *were* motivated by the health threat of COVID-19 to deliberately plan on quitting smoking (Jackson et al., 2021; Kaufman et al., 2018), however, the stress of the pandemic or being at home more often (avoiding public smoking restrictions) led to rapid decisions to actually smoke more in the moment.

### The present study

In response to mixed findings in previous research and contradictions with health behavior theory, the current study investigates how risk perceptions, regulatory attitudes, and cessation intentions (i.e., quitting intentions) among adults residing in Central California shifted amidst the COVID-19 pandemic lockdown. Although risk perceptions regarding COVID-19 among smokers have been assessed (Nyman et al., 2021; Yingst et al., 2021), to our knowledge, no studies have examined risk perceptions and regulation attitudes towards tobacco and tobacco-related products in the context of the COVID-19 pandemic. The dichotomy between people who had not experienced COVID-19 and those who had was established by the California COVID-19 lockdown in 2020, which signified a period where the knowledge and awareness of COVID-19 as a serious and highly contagious illness became conspicuous among the general population. The present study examined risk perceptions, regulatory attitudes, and cessation intentions towards tobacco-related products between these two groups: people who not yet experienced a major public health crisis, the COVID-19 pandemic, and those who had.

The specific aims of the study were to: (1) examine the relationships between experiencing the COVID-19 lockdown, smoking behaviors, and tobacco-related perceptions/attitudes; and (2) evaluate whether there were differences in smoking cessation intentions for those who did and did not experience the COVID-19 lockdown. Considering that smokers are at an increased risk of developing severe COVID-19 symptoms, and building on the HBM, we predict that individuals after the lockdown will exhibit greater awareness of the risks associated with tobacco use and show more support for tobacco regulation measures than those before the lockdown. This anticipated change is expected to be more pronounced among daily smokers, given their heightened vulnerability to severe COVID-19 complications. Regarding the second objective, based on the HBM and supported by previous research (Klemperer et al., 2020; Nyman et al., 2021; Yingst et al., 2021), we anticipate there will be higher intentions to cease smoking among post-lockdown individuals compared to pre-lockdown individuals.

## Methods

### Participants and procedure

Participants were recruited through targeted social media advertising on the social media platform, Facebook. Brief advertisements, with pictures and text inviting adults to share their thoughts about tobacco, were shown to Facebook account holders who resided in the 11-county area of Central California (e.g., counties included San Joaquin, Stanislaus, Fresno, Kern, etc.). Interested participants were then directed to a screening survey online hosted by Qualtrics. The present study analyzed a subsample (n = 2550) derived from the total sample (n = 3161). Participants were excluded for several reasons: extreme outliers in survey completion time (i.e., beyond 2 standard deviations from the mean), repeat responses, and missing or unclear responses on key variables, including tobacco risk perceptions and regulatory attitudes. Further exclusions included individuals under 18 years old and those who identified as “other” gender, resulting in a final sample of 2550 participants. This sample included 1349 individuals who completed the survey in March 2020 and 1201 individuals who completed the survey in May 2020. The survey was administered via Qualtrics. Inclusion criteria included age over 18 years, residence in Central California, and English literacy. Participants were compensated with a $5 gift certificate upon completion of the survey. The study protocol was approved by the Institutional Review Board (IRB) at the University of California, Merced (UCM2019-63). Prior to participation, all individuals provided electronic informed consent in accordance with institutional and ethical guidelines.

### Measures

#### Dependent variables

##### Risk perceptions

Participants were asked to rate how likely a typical smoker will become addicted to cigarettes, harm their own health, and harm the health of others on a 5-point Likert scale ranging from *Very Unlikely* (1) to *Very Likely* (*5*).

##### Regulatory attitudes

A composite was created with 7 items to measure attitudes towards tobacco regulations. The Cronbach’s Alpha was α = 0.75. Observations that included responses to at least 5 of the items were included and all of the items were measured on a 5-point Likert scale ranging from *Strongly agree (1*) to *Strongly disagree (5)* or *Definitely yes (1)* to *Definitely no (5)*. The slight differences in response options were for grammatical reasons; there is no difference in the content or directionality of the items. Example items include: “Adults should be allowed to smoke if they want to” (*Strongly agree* to *Strongly disagree*), “Should cigarette smoking be allowed in apartments/condos?” (*Definitely yes* to *Definitely no*) and, “Should cigarette smoking be allowed in sports venues/concerts?” (*Definitely yes* to *Definitely no)*. Higher scores represent higher disagreement with the statements and questions, indicating more favorability towards tobacco regulations.

##### Cessation Intentions

To measure participants’ intentions to quit smoking in the future, they were asked the following questions: “Are you planning to quit cigarettes in the next 6 months?” and “Are you planning to quit cigarettes in the next 30 days?” with response options of “yes” or “no.”

#### Independent variables

##### COVID-19 state lockdown

Lockdown status was a binary measure–participants who completed the survey in early March 2020 were not subject to the lockdown orders (pre-lockdown), whereas participants in the May 2020 survey had experienced the lockdown for over 2 months (post-lockdown). Although the COVID-19 virus was in California by early March, case numbers were low, it was not yet considered public health emergency, and daily life continued. The California stay at home order, which entailed all non-essential businesses to close and people were encouraged to avoid going out or gathering with others, was initiated March 19, 2020. Thus, the COVID-19 landscape in May was quite different–COVID-19 was declared a national health emergency (Centers for Disease Control, 2020), and people had been in the lockdown for over 2 months. These factors highlight the major COVID-19 experience difference between the groups that is being investigated.

##### Smoking-related behaviors

For smoking frequency, participants reported on how many of the past 30 days they smoked cigarettes. Those who took the survey in March reported on their smoking behaviors in February 2020 (i.e., before COVID-19 was declared a pandemic) and participants taking the survey in May reported on their smoking behaviors in April 2020 (i.e., during lockdown). Reponses were grouped into the following categories: 1–8 days, 9–21 days, 22–29 days, or daily.

##### Demographics

Participants were asked basic demographic questions, including age, gender identity (male; female), race/ethnicity (White; Black; American Indian/Alaska Native; Mixed; Other; Hispanic; Non-Hispanic), income (USD 0–USD 50,000; USD 51,000–USD 75,000; USD 76,000–USD 100,000; ≥ USD 101,000), and education (high school or less; some college/associates degree; bachelor’s degree or higher).

### Statistical analyses

Analyses of covariances (*ANCOVAs*) were performed to analyze the main effects and interaction effects between lockdown experience and smoking frequency on smoking risk perceptions and regulatory attitudes. All models adjusted for age, gender, ethnicity, race, income, and education. ANCOVA was chosen because of its ability to detect and estimate interactions, particularly through predictive margins (PM). PMs produce an adjusted mean for each of the lockdown status and smoking frequency groups and generated margin plots.

Multivariable logistic regressions were run to examine the relationship between lockdown experience and smoking cessation intentions. Only significant demographic variables were included in the final models. The model assessing cessation intentions in the next 6 months adjusted for gender, age, race, ethnicity, income, and education; the model assessing cessation intentions in the next 30 days adjusted for age only. Smoking frequency was not included because preliminary logistic regression analyses suggested no significant interaction between smoking frequency and cessation intentions between pre and post lockdown individuals.

## Results

### Study samples

The pre-lockdown and post-lockdown samples had comparable demographic proportions (Table 1). The average age of participants was 30.29 years (*SD* = 5.91). Most participants were either former or current cigarette smokers (96.29%), male (62.56%), non-Hispanic White (70.82%) and had some college education (31.60%) or a bachelor’s degree or higher (51.25%). The majority of the sample resided in a household where the income level was between USD26,000 and USD100,000 (USD 26,000–$50,000 = 23.45%; USD 51,000–$75,000 = 33.36%; USD 76,000–USD 100,000 = 25.90%).

**Table 1.** Overall sample characteristics, n = 2550.

Measures	Pre-lockdown *n* (%)^a^	Post-lockdown *n* (%)^a^	Total *n* (%)^a^
*n*	1349 (52.90%)	1201 (47.10%)	2550 (100%)
Smoked 100 cigarettes in lifetime	1141 (95.16%)	1038 (97.56%)	2179 (96.29%)
Male	889 (66.00%)	705 (58.70%)	1594 (62.56%)
23–35 years old	1008 (74.72%)	999 (83.18%)	2007 (78.71%)
Race/ethnicity^b^			
Hispanic	293 (21.72%)	144 (11.99%)	437 (17.14%)
NH White	904 (67.02%)	902 (75.10%)	1806 (70.82%)
NH Black	66 (4.89%)	83 (6.91%)	149 (5.84%)
NH NA	11 (0.82%)	11 (0.92%)	22 (0.86%)
NH Asian	50 (3.71%)	40 (3.33%)	90 (3.53%)
NH mixed/other	25 (1.85%)	21 (1.75%)	46 (1.80%)
At least 1 child under 18 in household	893 (66.30%)	646 (53.79%)	1539 (60.40%)
Education			
High school or less	182 (13.52%)	244 (21.44%)	426 (17.15%)
Some college/associates degree	498 (37.00%)	287 (25.22%)	785 (31.60%)
Bachelor’s degree or higher	666 (49.41%)	607 (53.34%)	1273 (51.24%)
Income			
≤ USD $50,000	345 (26.56%)	315 (26.25%)	660 (26.06%)
USD $51,000–USD $75,000	430 (32.26%)	415 (34.58%)	845 (33.36%)
USD $76,000–USD $100,000	341 (25.58%)	315 (26.25%)	656 (25.90%)
≥ USD $101,000	217 (16.28%)	155 (12.92%)	372 (14.68%)

^a^Due to missing data, some categories do not add to 100% (*n* = 2550).

^b^NH = non-hispanic; NA = Native American; USD = United States Dollar.

### Risk perceptions

There were significant differences between lockdown exposure groups on perceptions about cigarette smoking addiction (*p* < .000; *d* = 0.35), smoking harming oneself (*p* = .0018; *d* = 0.32), and secondhand smoke health threats (*p =* .0021; *d* = 0.29). PMs indicated that pre-lockdown participants believed in a higher likelihood that cigarettes: lead to addiction (PM, 4.30; 95% CI, 4.24–4.36); are harmful for one’s own health (PM, 4.10; 95% CI, 4.01–4.13); and harm others’ health (PM, 4.00; 95% CI, 3.93–4.05) compared to post-lockdown participants (addiction PM, 3.96; 95% CI*,* 3.90–4.03; harm own health PM, 3.84; 95% CI, 3.77–3.91; harm others’ health PM, 3.76; 95% CI, 3.69–3.82).

**Figure 1.**
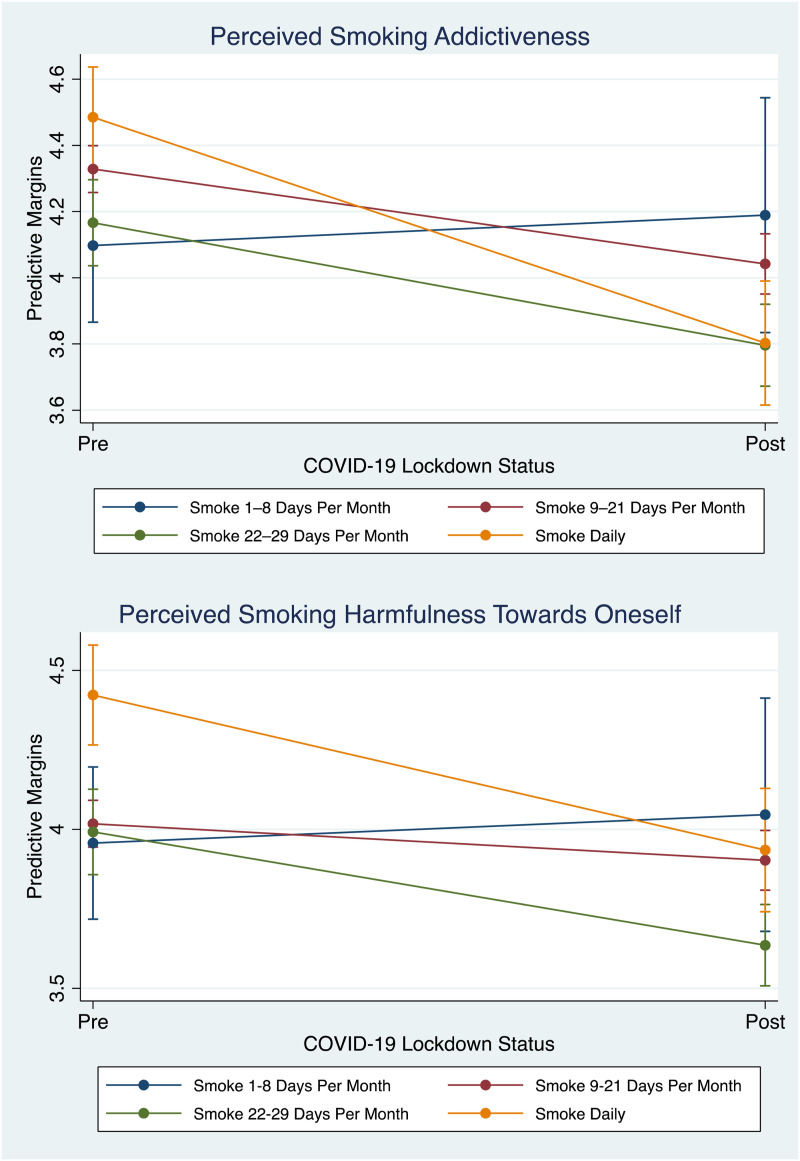
Margins plots demonstrating interaction effects between lockdown status and smoking frequency on risk perceptions towards smoking cigarettes, *n* = 1926.

Further analyses revealed an interaction between the effects of lockdown experience and smoking frequency on perceptions of cigarette addictiveness, *F* (3,21) = 4.15, *p* = .0061, and perceptions of harmfulness of cigarettes towards self, *F* (3,21) = 3.76, *p* = .0105, (Figure 1). Table 2 presents the PM analysis, revealing distinct patterns across different groups of smokers. Specifically, daily smokers post-lockdown perceived cigarettes to be less addictive (PM = 3.80, 95% CI, 3.62–3.99) and less harmful (PM = 3.93, 95% CI, 3.74–4.13) compared to their pre-lockdown counterparts (PM for addictiveness = 4.49, 95% CI, 4.33–4.64); PM for harmfulness = 4.42, 95% CI, 4.27–4.58). Cohen’s *d* estimates showed a medium to large effect size for perceptions of addictiveness (*d* = 0.70) and a medium effect size for perceptions of harmfulness (*d* = 0.60).

**Table 2.** Predictive margins analysis across pre and post lockdown groups.

Item	Smoked 1–8 days per month		Cohen’s *d*	Smoked daily		Cohen’s *d*
	Pre-lockdown	Post-lockdown		Pre-lockdown	Post-lockdown	
	PM (95% *CI*)	PM (95% *CI*)		PM (95% *CI*)	PM (95% *CI*)	
Perception of smoking addictiveness (*n* = 1926)	4.10* (3.87–4.33)	4.19* (3.83–4.54)	0.04	4.49* (4.33–4.64)	3.80* (3.62–3.99)	0.70
Perception of smoking harmfulness towards oneself (*n* = 1926)	3.96* (3.72–4.20)	4.05* (3.68–4.41)	0.15	4.42* (4.27–4.58)	3.93* (3.74–4.13)	0.60
Favorability towards tobacco regulation (*n* = 1923)	2.59* (2.42–2.76)	2.63* (2.37–2.90)	0.12	2.50* (2.38–2.90)	2.93* (2.79–3.08)	0.62

*Note*. PM = Predictive Margin.

*p < .001.

Conversely, participants who reported smoking 1–8 days per month post-lockdown perceived cigarettes as more addictive (PM = 4.19, 95% CI, 3.83–4.54) and more harmful (PM = 4.05, 95% CI, 3.68–4.41) than their pre-lockdown counterparts (PM for addictiveness = 4.10, 95% CI, 3.87–4.33); PM for harmfulness = 3.96, 95% CI, 3.72–4.20). Cohen’s *d* values for this group were small (*d* = 0.04 for addictiveness and *d* = 0.15 for harmfulness).

### Tobacco regulatory attitudes

Simple main effect analysis showed that there was a significant difference regarding tobacco regulation attitudes between pre- and post-lockdown individuals, *p* < .000, *d* = 0.20. Post-lockdown respondents had higher favorability towards regulations (PM, 2.80; 95% CI, 2.75–2.85) than pre-lockdown respondents (PM, 2.50; 95% CI, 2.45–2.54).

**Figure 2.**
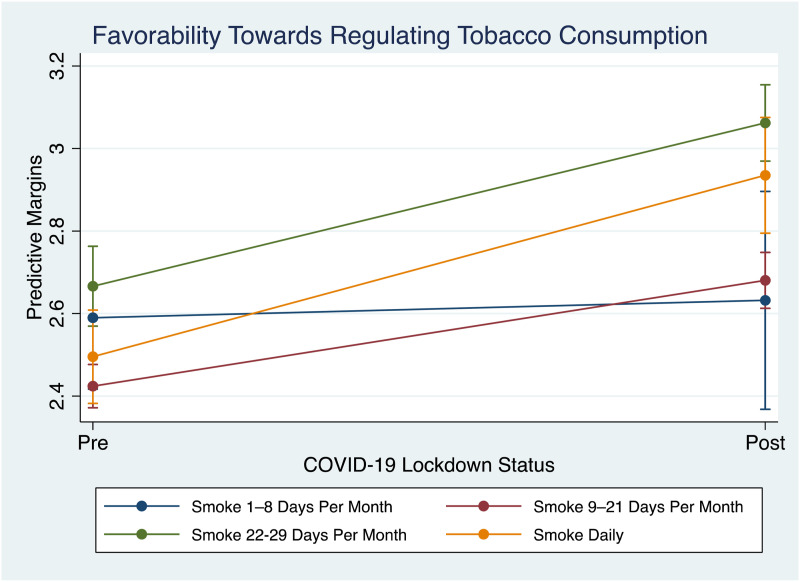
Margins plot demonstrating interaction effects between lockdown status and smoking frequency on tobacco regulatory attitudes, *n* = 1923.

There was a statistically significant interaction between the effects of lockdown experience and smoking frequency on tobacco regulation attitudes (Figure 2). Specifically, Table 2 highlights that among daily smokers, those in the post-lockdown phase demonstrated a higher approval of tobacco regulations (PM, 2.93 95% CI, 2.79–3.08) compared to their pre-lockdown counterparts (PM, 2.50; 95% CI, 2.38–2.61); Similarly, individuals who reported smoking 1–8 days per month in the post-lockdown period exhibited marginally increased favorability towards tobacco regulations (PM, 2.63 95% CI, 2.37–2.90) relative to the pre-lockdown phase (PM, 2.59; 95% CI, 2.42–2.76). Cohen’s *d* estimates indicate a medium effect size (d = 0.62) for the shift in regulatory attitudes among daily smokers from the pre-lockdown to the post-lockdown period. For individuals smoking 1–8 days per month, the effect size was small (d = 0.12).

### Cessation intentions

A subset sample (*n* = 2187) consisting of only current smokers was used to analyze cigarette cessation intentions. This sample includes individuals who have smoked a cigarette in their life before, have smoked at least 100 cigarettes, and have smoked at least once in the past 30 days (Supplemental Table 2). Logistic regression was used to examine the relation between whether COVID-19 lockdown was experienced and intentions to quit smoking cigarettes in: (1) the next 6 months; and (2) the next 30 days (Supplemental Table 1). The odds of intentions to quit smoking cigarettes in the next 6 months were 2.11 times greater for post-lockdown individuals than pre-lockdown individuals (*n* = 1830; *p* = .000; 95% CI, 1.68–2.63) and 1.61 times greater for intentions to quit smoking in the next 30 days for post-lockdown individuals than pre-lockdown individuals (*n* = 790; *p* = .020; 95% CI, 1.08–2.41).

## Discussion

The results of this study imply that a widespread public health crisis can exert a notable influence on health attitudes and perceptions. By acknowledging environmental contexts, it is evident that further complexities underlie the dynamic between smoking behaviors and related attitudes and perceptions. Our hypothesis that post-lockdown individuals would have higher tobacco-related risk perceptions compared to pre-lockdown individuals did not hold; the current study findings suggest overall lower tobacco risk perceptions among people who had experienced the COVID-19 lockdown. In accordance with our other predictions, post-lockdown individuals reported increased endorsement of tobacco regulations and greater intentions to quit smoking than those who had not experienced the COVID-19 lockdown.

However, not all participants in the post-lockdown group had lower tobacco risk perceptions (i.e., perceived tobacco to be less addictive and harmful) as this was dependent on smoking frequency. Counter to our hypothesis, post-lockdown *daily* smokers were more likely to report perceptions that smoking is not as addictive and harmful than their pre-lockdown counterparts, but post-lockdown participants who smoked *infrequently* (1–8 days per month) reported perceptions that smoking was more addictive and harmful than their pre-lockdown counterparts. In other words, it seems the perceived risks associated with cigarette smoking shifted among both daily and infrequent smokers in response to the COVID-19 pandemic and subsequent lockdown. Notably, the magnitude of change in risk perceptions was significantly greater among daily smokers when comparing the period before and after the lockdown, as opposed to the more modest change observed among those who smoke less frequently. This effect size difference indicates a pronounced difference in how the pandemic influenced smoking-related risk perceptions across these two groups.

This suggests a complex interplay where risk perceptions towards smoking may have varied during the lockdown—rising for some while falling for others—based on their smoking behavior (McCoy et al., 1992). In particular, this shift in perceptions may be a consequence of the varied smoking behavior changes amidst the pandemic (Gonzalez et al., 2021; Nyman et al., 2021; Vogel et al., 2021; Yingst et al., 2021). Given that our sample predominantly consists of current or former smokers (96.29%), it is plausible that their risk perceptions and regulatory attitudes were closely tied to their smoking actions, which could have been particularly influenced by the pandemic’s unique pressures and stressors. Participants’ smoking frequencies were self-reported for the past 30 days, with pre-lockdown data reflecting February 2020 behavior and post-lockdown data reflecting April 2020 behavior, at the lockdown’s height. Influences such as COVID-19’s severity (Karanasos et al., 2020) and increased health awareness (Bruine De Bruin and Bennett, 2020; Pu et al., 2020) might have led to reduced smoking for some, while heightened stress or isolation could have prompted others to smoke more (Gonzalez et al., 2021; Smith et al., 2020; Zyl-Smit et al., 2020). Given that the post-lockdown sample may have either increased or decreased their smoking frequency due to factors particular to the COVID-19 pandemic, it is reasonable to expect that their recent behavioral changes would have been reflected in their risk perceptions.

This analysis suggests that individuals who reduced smoking during the pandemic likely did so due to a heightened health consciousness and an awareness of COVID-19’s risks, as theorized by the HBM. Perhaps these individuals who reduced their smoking behaviors in response to the pandemic are the ones driving the increased risk perceptions among post-lockdown non-daily smokers. Conversely, perhaps those shifted to daily smoking during the pandemic are driving the decreased risk perceptions among post-lockdown daily smokers. Dual-process models (Gerrard et al., 2008) argue that people can pursue harmful health behaviors despite risk awareness due to a conflict between their deliberate, risk-acknowledging thought processes and their impulsive, pleasure-seeking judgment system, particularly under stress. Considering the widespread awareness of COVID-19’s severity and the universally recognized risks associated with the virus, it stands to reason that individuals who increased their smoking to daily levels during the pandemic were also cognizant of the health hazards of smoking. Cognitive dissonance theory (Festinger, 1962), can be applied to explain that the discomfort arising from the clash between knowing the dangers of smoking and engaging in increased smoking might have led these individuals to adjust their risk perceptions as a coping mechanism. Specifically, some smokers may have rationalized their increased smoking during the pandemic as a temporary behavior, thereby reducing its perceived addictiveness and harmfulness with the intention of returning to previous levels post-pandemic.

Thus, this subgroup of individuals might have reported lower risk perceptions to cope with this dissonance, but in line with their deliberate thinking system, still demonstrate greater support for tobacco regulation compared to pre-lockdown daily smokers. Considering the weak correlations between risk perceptions and regulatory attitudes found in the current study (Supplemental Table 3), it is possible that that increased regulatory attitudes among post-lockdown individuals were independent of risk perceptions and were, rather, related to another factor at the time. For instance, the COVID-19 lockdown period was characterized by a series of unprecedented regulations. Perhaps this encouraged people to be more accepting of other health regulation policies, particularly regarding tobacco.

### Limitations

The authors of this paper recognize the theoretical implications of the results and that further research may be required to address study limitations. The primary issue pertains to the underlying assumptions that explain the observed outcomes, namely, that the post-lockdown sample included a substantial number of individuals with recent smoking behavior changes attributable to the pandemic and lockdown measures. However, previous studies have reported significant fluctuations in smoking behaviors during and after lockdowns, indicating that the current study’s assumptions may be valid. Moving forward, it is crucial to explore whether the timing and motivations behind smoking behavior changes during the pandemic contributed to the observed disparities in perceptions and attitudes between daily and non-daily smokers.

The number of participants who reported intentions to quit within the next month was small, limiting the generalizability of the findings to the broader population of smokers. The sample was drawn from one region (California), which is largely rural, but also has some suburban and urban areas, which may limit generalizability to other rural and urban areas in the state and country.

Additionally, the study was carried out during a specific period of the COVID-19 pandemic, which has undergone significant transformations since Spring 2020. For example, the scientific and medical community has gained a deeper understanding of the COVID-19 virus and vaccines and treatments have been developed. As such, while this study provides insight into how perceptions and attitudes towards health-related behaviors can change during a widespread public health crisis, it is possible that these shifts may not be persistent.

However, insights from our research remain important to prevention and intervention strategies. Results demonstrating heightened tobacco-related risk perceptions among certain people who had experienced the pandemic presents an opportunity for public health initiatives to capitalize on this shift by reinforcing the connection between smoking, respiratory well-being, and increased vulnerability to viral infections, thus building on the elevated health awareness prompted by the pandemic. Additionally, the finding that perceptions and attitudes towards tobacco differed among various types of smokers provides enduring insights for smoking cessation strategies, underscoring the importance of tailoring cessation efforts to effectively address the distinct motivations and challenges faced by different types of smokers.

## Conclusion

The results of this study shed light on the heterogeneity of tobacco perceptions and attitudes across different types of smokers and underscore the impact of socioenvironmental events on tobacco-related beliefs, particularly in rural and understudied populations. Specifically, this paper highlights the intricate and nuanced relationship between risk perceptions and health behaviors, especially in the context of a public health crisis. While the immediate crisis of COVID-19 may have subsided, the insights gained offer a unique and valuable perspective on how to approach smoking prevention and intervention in the future. These findings underscore the importance of flexible, context-sensitive public health strategies that can adapt to changing global health landscapes and could have significant implications for refining cessation efforts to better serve current smokers.

## Supplemental Material

**Supplemental Material -** Tobacco harm perceptions, regulatory attitudes, and cessation intentions before and after the COVID-19 lockdown in California Supplemental Material for Tobacco harm perceptions, regulatory attitudes, and cessation intentions before and after the COVID-19 lockdown in California by Natalie R Beylin, Anna V Song and Anna E Epperson in Health Psychology Open.

## References

[bibr1-20551029241306095] Alcala E Cisneros R Capitman JA (2018) Health care access, concentrated poverty, and pediatric asthma hospital care use in California’s San Joaquin Valley: a multilevel approach. Journal of Asthma 55(11): 1253–1261. DOI: 10.1080/02770903.2017.1409234. 29261336

[bibr2-20551029241306095] Alizadehsani R Alizadeh Sani Z Behjati M , et al. (2021) Risk factors prediction, clinical outcomes, and mortality in COVID-19 patients. Journal of Medical Virology 93(4): 2307–2320. DOI: 10.1002/jmv.26699. 33247599 PMC7753243

[bibr3-20551029241306095] Bruine de Bruin W Bennett D (2020) Relationships between initial COVID-19 risk perceptions and protective health behaviors: a national survey. American Journal of Preventive Medicine 59(2): 157–167. DOI: 10.1016/j.amepre.2020.05.001. 32576418 PMC7242956

[bibr4-20551029241306095] Buettner-Schmidt K Miller DR Maack B (2019) Disparities in rural tobacco use, smoke-free policies, and tobacco taxes. Western Journal of Nursing Research 41(8): 1184–1202. DOI: 10.1177/0193945919828061. 30774036 PMC6613179

[bibr7-20551029241306095] Center for Disease Control (2020) COVID Data Tracker. Atlanta, Georgia: Centers for Disease Control and Prevention. https://covid.cdc.gov/covid-data-tracker.

[bibr8-20551029241306095] Centers for Disease Control and Prevention (2021) Fast Facts. Atlanta, Georgia: Centers for Disease Control and Prevention. https://www.cdc.gov/tobacco/data_statistics/fact_sheets/fast_facts/index.html.

[bibr9-20551029241306095] Centers for Disease Control and Prevention (2022) People with Certain Medical Conditions. Atlanta, Georgia: Centers for Disease Control and Prevention. https://www.cdc.gov/coronavirus/2019-ncov/need-extra-precautions/people-with-medical-conditions.html. COVID-19.

[bibr10-20551029241306095] Coffman J Geyn I Himmerick A (2017) Californoa’s primary care workforce: Current supply, characteristics, and pipeline of trainees. Healthforce Center at UCSF. Research Report. Available at: https://pmfmd:.com/wp-content/uploads/2019/12/PMF-2018-Report-UCSF-Healthforce-Center_exec.-Summary-report-on-CA-MD-NP-DO.pdf.

[bibr11-20551029241306095] Cummings KM Proctor RN (2014) The changing public image of smoking in the United States: 1964–2014. Cancer Epidemiology, Biomarkers & Prevention 23(1): 32–36. DOI: 10.1158/1055-9965.EPI-13-0798. PMC3894634 24420984

[bibr12-20551029241306095] Doogan NJ Roberts ME Wewers ME , et al. (2017) A growing geographic disparity: rural and urban cigarette smoking trends in the United States. Preventive Medicine 104: 79–85. DOI: 10.1016/j.ypmed.2017.03.011. 28315761 PMC5600673

[bibr13-20551029241306095] Festinger L (1962) A Theory of Cognitive Dissonance. Redwood City, CA: Stanford University Press.

[bibr14-20551029241306095] Frieden TR Mostashari F Kerker BD , et al. (2005) Adult tobacco use levels after intensive tobacco control measures: New York city, 2002–2003. American Journal of Public Health 95(6): 1016–1023. DOI: 10.2105/AJPH.2004.058164. 15914827 PMC1449302

[bibr15-20551029241306095] Gerrard M Gibbons FX Houlihan AE , et al. (2008) A dual-process approach to health risk decision making: the prototype willingness model. Developmental Review 28(1): 29–61. DOI: 10.1016/j.dr.2007.10.001.

[bibr16-20551029241306095] Gilpin EA Emery SL Farkas AJ , et al. (2001) The California Tobacco Control Program: A Decade of Progress, Results from the California Tobacco Survey, 1990-1999: (612172007-001). Washington, DC: American Psychological Association. DOI: 10.1037/e612172007-001.

[bibr17-20551029241306095] Gilpin EA Lee L Pierce JP (2004) Changes in population attitudes about where smoking should not be allowed: California versus the rest of the USA. Tobacco Control 13(1): 38–44. DOI: 10.1136/tc.2003.004739. 14985593 PMC1747831

[bibr18-20551029241306095] Gonzalez M Epperson AE Halpern-Felsher B , et al. (2021) Smokers are more likely to smoke more after the COVID-19 California lockdown order. International Journal of Environmental Research and Public Health 18(5): 2582. DOI: 10.3390/ijerph18052582. 33807503 PMC7967350

[bibr19-20551029241306095] Gülsen A Yigitbas BA Uslu B , et al. (2020) The effect of smoking on COVID-19 symptom severity: systematic review and meta-analysis. Pulmonary Medicine 2020: e7590207. DOI: 10.1155/2020/7590207. PMC7499286 32963831

[bibr20-20551029241306095] Hyland A Barnoya J Corral JE (2012) Smoke-free air policies: past, present and future. Tobacco Control 21(2): 154–161. DOI: 10.1136/tobaccocontrol-2011-050389. 22345239

[bibr21-20551029241306095] Jackson SE Brown J Shahab L , et al. (2021) COVID-19, smoking and inequalities: a study of 53 002 adults in the UK. Tobacco Control 30(e2): e111–e121. DOI: 10.1136/tobaccocontrol-2020-055933. 32826387

[bibr22-20551029241306095] Karanasos A Aznaouridis K Latsios G , et al. (2020) Impact of smoking status on disease severity and mortality of hospitalized patients with COVID-19 infection: a systematic review and meta-analysis. Nicotine & Tobacco Research 22(9): 1657–1659. DOI: 10.1093/ntr/ntaa107. 32564072 PMC7337737

[bibr23-20551029241306095] Kaufman AR Persoskie A Twesten J , et al. (2018) A review of risk perception measurement in tobacco control research. Tobacco Control 29: s50–s58. DOI: 10.1136/tobaccocontrol-2017-054005, tobaccocontrol-2017-054005. 29432136

[bibr24-20551029241306095] Klemperer EM West JC Peasley-Miklus C , et al. (2020) Change in tobacco and electronic cigarette use and motivation to quit in response to COVID-19. Nicotine & Tobacco Research 22(9): 1662–1663. DOI: 10.1093/ntr/ntaa072. 32343816 PMC7197523

[bibr25-20551029241306095] Li Y Luk TT Wu Y , et al. (2021) High perceived susceptibility to and severity of COVID-19 in smokers are associated with quitting-related behaviors. International Journal of Environmental Research and Public Health 18(20): 10894. DOI: 10.3390/ijerph182010894. 34682641 PMC8535969

[bibr26-20551029241306095] Matthews KA Croft JB Liu Y , et al. (2017) Health-related behaviors by urban-rural county classification—United States, 2013. MMWR Surveillance Summaries 66(5): 1–8. DOI: 10.15585/mmwr.ss6605a1. PMC5829834 28151923

[bibr27-20551029241306095] McCoy SB Gibbons FX Reis TJ , et al. (1992) Perceptions of smoking risk as a function of smoking status. Journal of Behavioral Medicine 15(5): 469–488. DOI: 10.1007/BF00844942. 1447758

[bibr28-20551029241306095] National Insitutes of Health (NIH) (2022) COVID-19 Was Third Leading Cause of Death in the United States in Both 2020 and 2021. Bethesda, MD: National Institutes of Health (NIH). https://www.nih.gov/news-events/news-releases/covid-19-was-third-leading-cause-death-united-states-both-2020-2021.

[bibr30-20551029241306095] Nyman AL Spears CA Churchill V , et al. (2021) Associations between COVID-19 risk perceptions and smoking and quitting behavior among U.S. adults. Addictive Behaviors Reports 14: 100394. DOI: 10.1016/j.abrep.2021.100394. 34869823 PMC8626346

[bibr31-20551029241306095] Patanavanich R Glantz SA (2020) Smoking is associated with COVID-19 progression: a meta-analysis. Nicotine & Tobacco Research 22(9): 1653–1656. DOI: 10.1093/ntr/ntaa082. 32399563 PMC7239135

[bibr32-20551029241306095] Pu B Zhang L Tang Z , et al. (2020) The relationship between health consciousness and home-based exercise in China during the COVID-19 pandemic. International Journal of Environmental Research and Public Health 17(16): 5693. DOI: 10.3390/ijerph17165693. 32781751 PMC7460040

[bibr33-20551029241306095] Reddy RK Charles WN Sklavounos A , et al. (2021) The effect of smoking on COVID-19 severity: a systematic review and meta-analysis. Journal of Medical Virology 93(2): 1045–1056. DOI: 10.1002/jmv.26389. 32749705 PMC7436545

[bibr34-20551029241306095] Rosenstock IM (1974) Historical origins of the health belief model. Health Education Monographs 2(4): 328–335. DOI: 10.1177/109019817400200403. 299611

[bibr35-20551029241306095] Smith L Jacob L Yakkundi A , et al. (2020) Correlates of symptoms of anxiety and depression and mental wellbeing associated with COVID-19: a cross-sectional study of UK-based respondents. Psychiatry Research 291: 113138. DOI: 10.1016/j.psychres.2020.113138. 32562931 PMC7258801

[bibr36-20551029241306095] Spada R Spada N Seon-Spada H (2019) Geographic disparities persist despite decline in mortality from IHD in California’s central valley 1999–2014. JRSM Cardiovascular Disease 8(2048004019866320): 2048004019866320. DOI: 10.1177/2048004019866320. 31391939 PMC6669834

[bibr37-20551029241306095] Stuber J Galea S Link BG (2008) Smoking and the emergence of a stigmatized social status. Social Science & Medicine 67(3): 420–430. DOI: 10.1016/j.socscimed.2008.03.010. 18486291 PMC4006698

[bibr41-20551029241306095] Vogel EA Henriksen L Schleicher NC , et al. (2021) Perceived susceptibility to and seriousness of COVID-19: associations of risk perceptions with changes in smoking behavior. International Journal of Environmental Research and Public Health 18(14): 7621. DOI: 10.3390/ijerph18147621. 34300072 PMC8307925

[bibr43-20551029241306095] Yingst JM Krebs NM Bordner CR , et al. (2021) Tobacco use changes and perceived health risks among current tobacco users during the COVID-19 pandemic. International Journal of Environmental Research and Public Health 18(4): 1795. DOI: 10.3390/ijerph18041795. 33673207 PMC7917755

