# Supplementary material for: Tobacco harm perceptions, regulatory attitudes, and cessation intentions before and after the COVID-19 lockdown in California
Source: Health Psychol Open. 2024 Dec 2;11:20551029241306095. doi: 10.1177/20551029241306095 (PMC11613243; doi:10.1177/20551029241306095)
Supplement: Supplemental Material - Tobacco harm perceptions, regulatory attitudes, and cessation intentions before and after the COVID-19 lockdown in California [file sj-pdf-1-hpo-10.1177_20551029241306095.pdf]

Supplemental Table 1

Multivariate Logistic Regression Analysis of Cigarette Smoking Cessation Intentions

	Intentions to Quit Next 30 Days			Intentions to Quit Next 6 Months		
Variable	Odds Ratio	Std. Error	95% conf. interval	Odds Ratio	Std. Error	95% conf. interval
Lockdown Status	1.61*	0.33	1.08–2.41	2.11**	0.24	1.68–2.63
Age	0.65**	0.10	0.48–0.89	0.89	0.08	0.72–1.01
Gender	-	-	-	1.10	0.13	0.87–1.39
Ethnicity	-	-	-	0.31**	0.05	0.23–0.42
Race	-	-	-			
NH Black	-	-	-	1.27	0.25	0.86–1.87
Other	-	-	-	2.39	0.47	1.63–3.52
Income						
USD $0–USD $50K	-	-	-	0.54**	0.10	0.38–0.77
USD $51K–USD $75K	-	-	-	0.76	0.12	0.55–1.05
USD $76K–USD $100K	-	-	-	0.87	0.14	0.64–1.19
Education						
High School	-	-	-	0.04**	0.10	0.02–0.08
Associates	-	-	-	0.27**	0.06	0.17–0.42
Bachelor’s	-	-	-	0.31**	0.07	0.20–0.47

**p* < .05. ***p* < .01.

Note. NH = Non-Hispanic. The following are the reference groups for the logistic regressions: NH White (Race); Over $101k (Income); Graduate or Professional Degree (Education).

Supplemental Table 2

Characteristics of Smoker Sample, n = 2187

Measures	Pre-Lockdown *n*(%)[a]	Post-Lockdown *n*(%)[a]	Total *n*(%)[a]
n	1147 (52.45%)	1040 (47.55%)	2187 (100%)
Male	795 (69.37%)	619 (59.52%)	1414 (64.65%)
23–35 years old	903 (76.63%)	863 (70.09%)	1766 (80.75%)
Ethnicity/Race			
Hispanic	192 (16.84%)	100 (9.63%)	292 (13.35%)
NH White	985 (86.25%)	661 (81.30%)	1646 (75.26%)
NH Black	71 (6.22%)	80 (9.84%)	151 (6.90%)
NH Mixed/Other	25 (1.85%)	72 (8.86%)	97 (4.44%)
Education			
High school or less	124 (10.83%)	231 (23.64%)	355 (16.23.15%)
Some college/associates degree	416 (36.33%)	252 (25.79%)	668 (30.54%)
Bachelor’s degree or higher	605 (52.83%)	494 (50.56%)	1099 (50.25%)
Income			
≤ USD $50,000	257 (22.52%)	297 (28.56%)	554 (25.33%)
USD $51,000–USD $75,000	370 (32.43%)	326 (31.35%)	696 (31.82%)
USD $76,000–USD $100,000	312 (27.34%)	266 (25.58%)	578 (26.43%)
≥ USD $101,000	202 (17.70%)	151 (14.52%)	353 (16.14%)

* NH = Non-Hispanic; USD = United States Dollar.

Supplemental Table 3

Correlations of Risk Perceptions and Regulatory Attitudes

Variable	*M*	*SD*	1	2	3
1. Perception of Smoking Addictiveness	4.13	.02			

2. Perception of Smoking Harmfulness Towards Oneself	3.97	.02	.27**		
3. Perception of Smoking Harmfulness Towards Others	3.91	.02	.29**	.33**	
4. Favorability Towards Tobacco Regulation	2.73	.02	-.21**	-.04*	-.05*

$^{*}p < .05$. $^{**}p < .01$.
